# An electrocatalytic route for transformation of biomass-derived furfural into 5-hydroxy-2(5*H*)-furanone†
†Electronic supplementary information (ESI) available. See DOI: 10.1039/c9sc00322c


**DOI:** 10.1039/c9sc00322c

**Published:** 2019-03-25

**Authors:** Haoran Wu, Jinliang Song, Huizhen Liu, Zhenbing Xie, Chao Xie, Yue Hu, Xin Huang, Manli Hua, Buxing Han

**Affiliations:** a Beijing National Laboratory for Molecular Science , CAS Key Laboratory of Colloid and Interface and Thermodynamics , CAS Research/Education Center for Excellence in Molecular Sciences , Institute of Chemistry , Chinese Academy of Sciences , Beijing 100190 , China . Email: songjl@iccas.ac.cn ; Email: hanbx@iccas.ac.cn; b School of Chemistry and Chemical Engineering , University of Chinese Academy of Sciences , Beijing 100049 , China

## Abstract

Development of efficient strategies for biomass valorization is a highly attractive topic. Herein, we conducted the first work on electrocatalytic oxidation of renewable furfural to produce the key bioactive intermediate 5-hydroxy-2(5*H*)-furanone (HFO). It was demonstrated that using H_2_O as the oxygen source and metal chalcogenides (CuS, ZnS, PbS, *etc.*) as electrocatalysts, the reaction could proceed efficiently, and the CuS nanosheets prepared in this work showed the best performance and provided high HFO selectivity (83.6%) and high conversion (70.2%) of furfural. In addition, the CuS electrocatalyst showed long-term stability. Mechanism investigation showed that furfural was oxidized to HFO *via* multistep reactions, including C–C cleavage, subsequent ring opening and oxidation, and intramolecular isomerization.

## Abstract 2

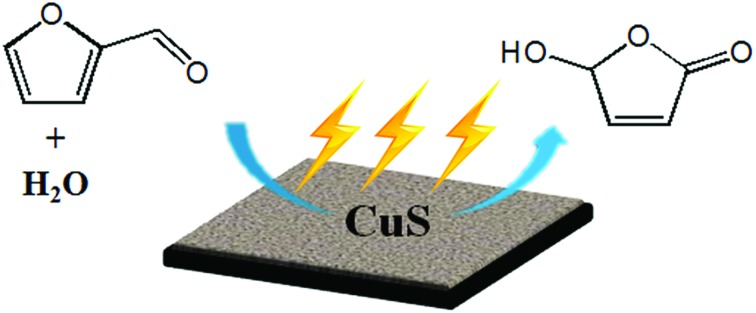
An electrocatalytic route was developed for the first time for conversion of biomass-derived furfural to bioactive 5-hydroxy-2(5*H*)-furanone over CuS nanosheets using H_2_O as the oxygen source.

## Introduction

With the gradual depletion of fossil fuel resources, utilization of renewable and abundant biomass to produce valuable chemicals and functional materials has become a very interesting field.^1^–^5^ In this regard, producing valuable chemicals using lignocellulose-derived furfural has attracted much attention.^6^–^8^

5-Hydroxy-2(5*H*)-furanone (HFO) is a key constituent in many biologically active compounds and intermediates.^9^ HFO can be synthesized by thermo-catalytic oxidation of high-cost 2-trialkylsilyloxyfurans with dimethyldioxirane as the oxidant.^10^ In comparison, direct oxidation of renewable furfural to produce high value-added HFO *via* thermo-oxidation is highly desired. However, it was very difficult to obtain HFO selectively from direct thermo-oxidation of furfural because HFO is easily transformed into maleic acid (MA) under thermocatalytic conditions.^11^ In current reports about furfural thermo-oxidation,^12^ HFO was only considered as the intermediate to synthesize MA, and no successful work has been reported with HFO as the target product from direct thermo-oxidation of furfural. On the other hand, photocatalysis has been applied in the oxidation of furfural or its derivatives (*i.e.*, furfuryl alcohol and furoic acid) to prepare HFO.^13^–^15^ Although progress has been made in photo-oxidation of furfural to HFO, the reliance on environmentally hazardous photosensitizers and their poor recyclability severely limited the applications of photocatalytic processes. Therefore, development of green, selective, and efficient routes for direct oxidation of furfural to HFO is highly desired but challenging.

Recently, electrocatalysis has sparked increasing interest due to its potential ability of solving thermodynamic and/or kinetic problems in thermocatalysis and photocatalysis. The pathway of electrocatalytic reactions is often different from those of thermocatalysis and photocatalysis. Electrocatalysis has been widely applied in the fields of hydrogen and oxygen evolution, CO_2_ conversion, and biomass transformation.^16^–^21^ Moreover, some active species (*e.g.*, hydroxyl radicals) for oxidation (especially in water treatment for waste oxidation) can be generated from H_2_O *via* electrocatalysis.^22^–^27^ Considering the advantages of electrocatalysis, it may be a promising route for direct oxidation of furfural to HFO using H_2_O as the oxygen source, but this has not been reported to date.

**Scheme 1:**
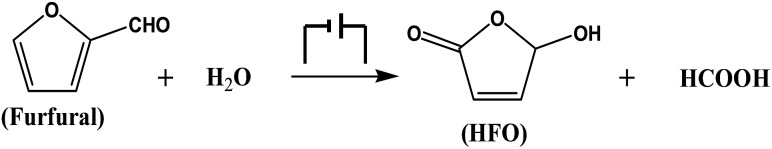
Electrocatalytic oxidation of furfural to HFO.

In this work, we proposed an electrocatalytic route for the oxidation of furfural to HFO and formic acid (Scheme 1). By employing simple metal chalcogenides (*i.e.*, CuS, ZnS, PbS, *etc.*) as electrocatalysts, furfural could be selectively oxidized to HFO using H_2_O as the oxygen source under ambient conditions in a ternary electrolyte consisting of triethylammonium nitrate ([Et_3_NH]NO_3_), acetonitrile (MeCN) and H_2_O, and the synthesized CuS nanosheets provided the best performance with a high HFO selectivity (83.6%) and high conversion (70.2%) of furfural. To the best of our knowledge, this is the first work to realize the oxidation of furfural to HFO *via* electrocatalysis.

## Results and discussion

### Synthesis and characterization of electrocatalysts

**Fig. 1:**
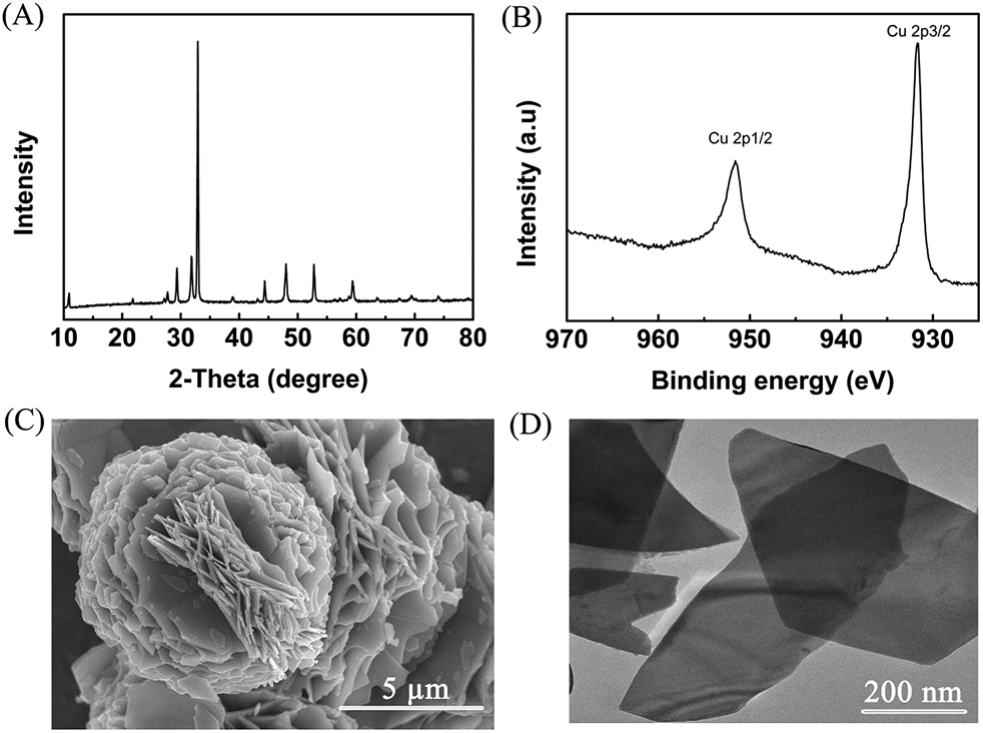
Characterization of the prepared CuS. (A) XRD pattern, (B) XPS of Cu 2p, (C) SEM image, and (D) TEM image.

Metal chalcogenides (*e.g.*, MoS_2_, Cu_2_S, ZnS, and CdS) have been considered as simple and efficient electrocatalysts owing to their unique electrical properties.^28^–^30^ Therefore, metal chalcogenides can be potentially applied as promising electrode materials for electro-oxidation. Initially, CuS was synthesized *via* a solvothermal process in a deep eutectic solvent consisting of PEG-200 and thiourea, and the detailed preparation procedure is described in the Experimental section. X-ray diffraction (XRD) examination was conducted to confirm the formation of CuS. The pattern in Fig. 1A shows the characteristic peaks of CuS (JCPDS: 65-3588), verifying that CuS could be indeed generated by the proposed route. Meanwhile, X-ray photoelectron spectra (XPS) showed the characteristic binding energies of Cu^2+^ (Cu 2p_3/2_ 931.7 and Cu 2p_1/2_ 951.6 eV) and S^2+^ (S 2p_3/2_ 161.6 and S 2p_1/2_ 162.7 eV) in the prepared material (Fig. 1B and S1†), further indicating the formation of CuS.^31^ Furthermore, as clearly shown in the scanning electron microscopy (SEM, Fig. 1C) and transmission electron microscopy (TEM, Fig. 1D) images, the prepared CuS had a nanosheet structure. For comparison, ZnS and PbS were also synthesized using a similar route to that for CuS preparation, and were characterized in detail by employing XRD, XPS, SEM and TEM (Fig. S2 and S3†).

### Performance of various electrocatalysts

**Fig. 2:**
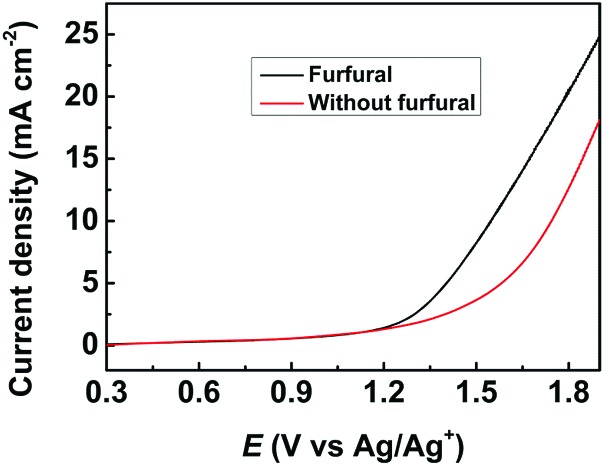
LSV measurements using the CuS/CP electrode for the electrochemical oxidation of furfural in the [Et_3_NH]NO_3_ (1.8 wt%)–MeCN–H_2_O (12.5 wt%) electrolyte.

The obtained metal chalcogenides (MC) were spread onto carbon paper (CP) as the electrodes (denoted as MC/CP) for the electrochemical oxidation of furfural. The electrocatalytic performance of the prepared MC/CP electrodes was initially evaluated by linear sweep voltammetry (LSV) measurements, which were conducted in an H-type cell containing three electrodes.^32^ In the experiments, a ternary electrolyte consisting of [Et_3_NH]NO_3_, MeCN and H_2_O was used as the anolyte, while aqueous H_2_SO_4_ solution (0.2 M) was employed as the catholyte. Meanwhile, the applied potential was swept from 0.3 to 1.9 V *vs.* Ag/Ag^+^ with a scan rate of 20 mV s^–1^. An obvious increase in current density could be found in the reaction systems with furfural in comparison to those without furfural (Fig. 2, S4–S8†), suggesting the occurrence of furfural electro-oxidation on the prepared MC/CP electrodes.

The performance of various prepared electrodes for the electrochemical oxidation of furfural was subsequently examined (Table 1). In the reaction process, the main liquid products were HFO, maleic acid (MA), and HCOOH along with a very small amount of maleic anhydride and 2(5*H*)-furanone (Scheme S1†). As shown in Scheme 1, HCOOH could be generated and the selectivity was above 95% in all experiments. Meanwhile the gaseous product was O_2_ from water electrolysis. As shown in Table 1, the prepared CuS/CP electrode (Table 1, entry 1) showed the best performance compared with ZnS/CP, PbS/CP, CdS/CP, MoS_2_/CP, and WS_2_/CP (Table 1, entries 2–6). As a comparison, copper oxide (CuO) was also employed as an electrocatalyst. Unfortunately, the CuO/CP electrode showed poorer electrocatalytic performance (Table 1, entry 7) than the prepared CuS/CP electrode (Table 1, entry 1). Moreover, the synthesized CuS nanosheets showed higher activity than commercial CuS composed of irregular particles (Fig. S9†). N_2_ adsorption–desorption isotherms (Fig. S10†) showed that no micropores existed in both the prepared CuS and the commercial one,^33^,^34^ and they had the same average pore diameter (3.82 nm). Meanwhile, the prepared CuS (18.0 m^2^ g^–1^) showed a higher BET surface area than the commercial one (12.7 m^2^ g^–1^). The higher surface area and the nanosheet structure of the synthesized CuS were helpful for the exposure of more active sites,^35^ and thus the activity of the synthesized CuS was higher than that of the commercial one. Additionally, when using bare carbon paper as the electrode (Table 1, entry 9), the furfural conversion and FE were much lower than those over the synthesized CuS/CP (Table 1, entry 1), implying the catalytic role of CuS in furfural oxidation.

**Table 1:** Electrochemical oxidation of furfural on different electrodes at an applied potential of 1.6 V (*vs*. Ag/Ag^+^) in the [Et_3_NH]NO_3_ (1.8 wt%)–MeCN–H_2_O (12.5 wt%) electrolyte (5.6 g) with 1 mmol of furfural by electrolysis for 7 h

Entry	Electrode	Conversion (%)^*a*^	Selectivity (%)^*a*^		FE (%)^*b*^
			HFO	MA	
1	CuS/CP	70.2	83.6	8.8	77.1
2	ZnS/CP	56.3	89.9	8.1	76.9
3	PbS/CP	63.7	83.5	9.4	78.3
4	CdS/CP	51.7	65.4	6.5	64.9
5	WS_2_/CP	69.9	68.7	9.4	69.2
6	MoS_2_/CP	61.4	64.2	6.9	63.4
7	CuO/CP	55.1	71.5	13.7	78.9
8^*c*^	CuS/CP	53.5	85.5	9.3	75.2
9	CP	23.8	72.3	10.1	61.8

^*a*^The conversion and selectivity were obtained from NMR examinations.

^*b*^FE represents the sum of faradaic efficiency of HFO and MA in this work.

^*c*^Commercial CuS was used as the electrocatalyst.

Electrochemical impedance spectroscopy (EIS) was carried out to get more interfacial information on the electrode/electrolyte interface (Fig. S11–S17 and Table S1†), and a simple equivalent circuit was used to fit the high and medium frequency data (Fig. S18†). As shown in Table S1,† CuS/CP showed the lowest charge transfer resistance (*R*_ct_, 34.51) among the tested electrodes (*i.e.*, CuS/CP, ZnS/CP, PbS/CP, CdS/CP, MoS_2_/CP, WS_2_/CP, and CuO/CP), indicating more facile electron transfer between the interface of CuS/CP and the anolyte. Meanwhile, the highest double layer capacitance (*C*_dl_) was achieved using the CuS/CP electrode (Table S1†), suggesting the higher charge density around CuS/CP. Both more facile electron transfer and higher charge density were beneficial for the generation of hydroxyl radicals (the active species for oxidation) from H_2_O electrolysis,^22^–^27^ and thus enhanced furfural oxidation on the CuS/CP electrode. Additionally, no obvious difference was found in solution resistance (*R*_s_) because the electrolyte with the same composition was employed for all reactions (Table 1), implying the negligible effect of *R*_s_ on the different activity of the used electrodes. The above results suggested that the prepared CuS/CP electrode was a superior electrode for the electrochemical oxidation of furfural to generate HFO owing to its lowest *R*_ct_ and highest *C*_dl_.

### Effect of various reaction parameters

**Fig. 3:**
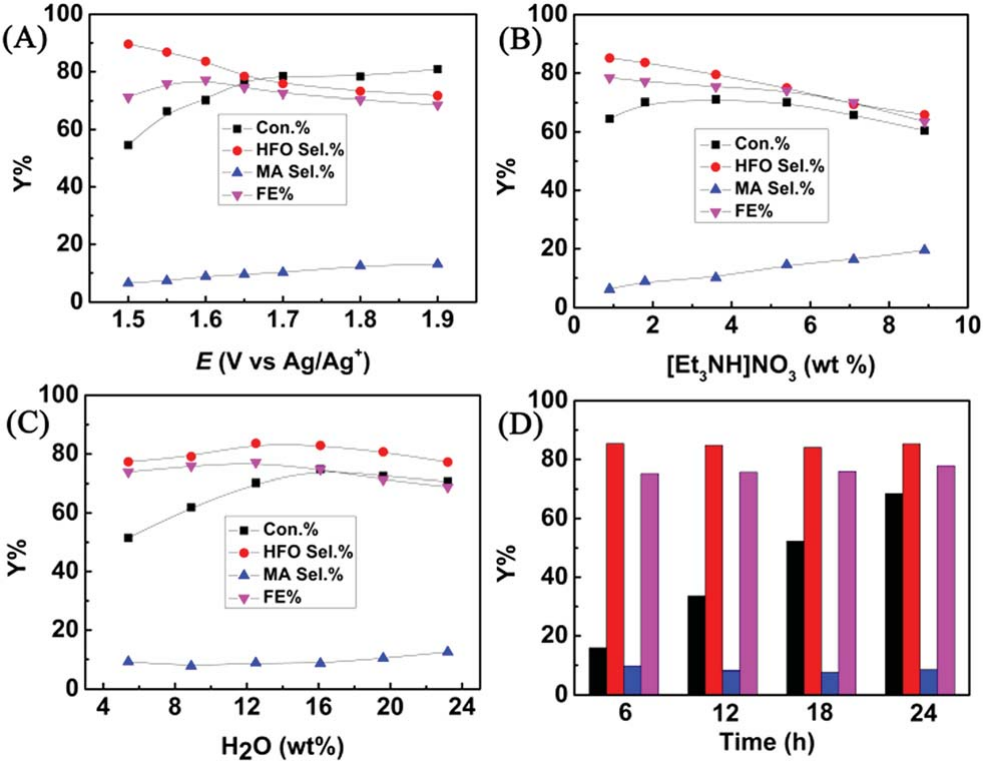
Effects of various parameters on the electrochemical oxidation of furfural to HFO over the CuS/CP electrode in the [Et_3_NH]NO_3_–MeCN–H_2_O electrolyte (5.6 g) with 1 mmol of furfural for 7 h. (A) Applied potential, (B) [Et_3_NH]NO_3_ concentration, (C) H_2_O concentration, and (D) long-term electrolysis for furfural oxidation using the CuS/CP electrode in the [Et_3_NH]NO_3_ (1.8 wt%)–MeCN–H_2_O (12.5 wt%) electrolyte (7.6 g) with 3 mmol of furfural at 1.6 V *vs.* Ag/Ag^+^ (black, red, blue, and magenta bars represent furfural conversion, HFO selectivity, MA selectivity, and FE, respectively).

The effect of applied potential on the electrochemical oxidation of furfural in the [Et_3_NH]NO_3_ (1.8 wt%)–MeCN–H_2_O (12.5 wt%) electrolyte was investigated. As shown in Fig. 3A, at a lower applied potential (1.5 V *vs.* Ag/Ag^+^), the HFO selectivity could reach 89.6%, but furfural conversion was low (54.6%). With the increase of the applied potential, the conversion of furfural gradually increased, while the HFO selectivity gradually decreased with increasing MA selectivity. The results indicated that a lower applied potential was not beneficial for furfural conversion because lower amounts of hydroxyl radicals were generated at a lower applied potential, and a higher potential resulted in formation of more hydroxyl radicals, which enhanced the conversion of HFO to MA. Control experiments using HFO as the reactant proved that the conversion of HFO increased with the increase in applied potential over the prepared CuS/CP electrode (Fig. S19†). In addition, a higher potential caused more oxygen evolution (a competitive reaction),^36^ resulting in the decrease of the faradaic efficiency. Therefore, 1.6 V (*vs.* Ag/Ag^+^) was selected as the optimal potential for the following investigations.

The composition of the anolyte could also significantly affect the electrochemical oxidation of furfural. In one aspect, the effect of [Et_3_NH]NO_3_ concentration was evaluated (Fig. 3B). A higher concentration of [Et_3_NH]NO_3_ could result in higher conductivity (Table S2†), which was beneficial for the generation of hydroxyl radicals. More hydroxyl radicals would cause further oxidation of HFO to MA, and thus the HFO selectivity decreased with increasing [Et_3_NH]NO_3_ concentration. Meanwhile, the FE and the furfural conversion also decreased at higher [Et_3_NH]NO_3_ concentrations because higher conductivity was also prone to enhance the oxygen evolution (a competitive reaction).^36^ Additionally, a lower concentration (0.9 wt%) of [Et_3_NH]NO_3_ was not beneficial for furfural conversion because lower conductivity was unhelpful for the formation of hydroxyl radicals. Therefore, by balancing the conversion and selectivity, 1.8 wt% [Et_3_NH]NO_3_ was the optimal concentration. In another aspect, H_2_O was indispensable for electrochemical oxidation because it could provide hydroxyl radicals as the active species for the oxidation.^22^–^27^ Therefore, the effect of H_2_O concentration in the anolyte was examined (Fig. 3C). The conversion of furfural increased with increase in the H_2_O amount within a certain concentration range (5.4–16.1 wt%) because H_2_O was helpful for the dissociation of ion pairs in [Et_3_NH]NO_3_ to enhance the generation of hydroxyl radicals, but the HFO selectivity reached the maximum at 12.5 wt% of H_2_O. However, a higher concentration of H_2_O (>12.5 wt%) was not beneficial for furfural oxidation to generate HFO because too much H_2_O provided more hydroxyl radicals, and thus enhanced the competitive oxygen evolution and further oxidation of HFO, resulting in a decrease in HFO selectivity and FE. Therefore, 12.5 wt% H_2_O was the suitable concentration in our electrocatalytic system. Finally, the long-term stability of the prepared CuS/CP electrode was examined (Fig. 3D). High HFO selectivity (85.3%) and high FE (77.8%) could be achieved with a reaction time of 24 h. Meanwhile, no obvious changes were found in the XRD patterns (Fig. S20†) and XPS spectra of Cu 2p and S 2p (Fig. S21†) of the virgin and used CuS/CP electrode, suggesting no other species was formed and no oxidation of the CuS surface occurred after electrocatalysis (24 h). The above results suggested the excellent long-term stability of the prepared CuS/CP electrode.

**Fig. 4:**
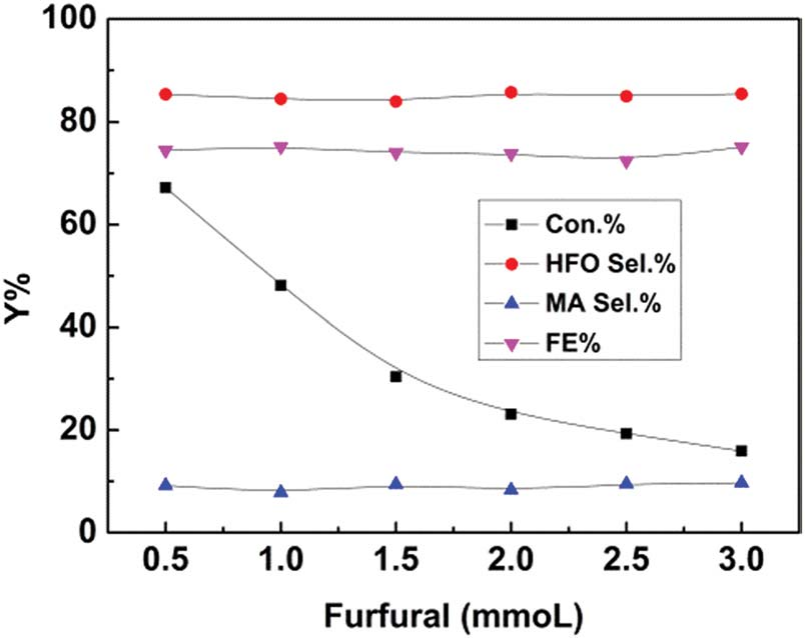
Effect of furfural concentration on the electrochemical oxidation of furfural over the CuS/CP electrode in the electrolyte (7.6 g) of [Et_3_NH]NO_3_ (1.8 wt%)–MeCN–H_2_O (12.5 wt%) for 6 h.

Furthermore, furfural concentration was also an important parameter affecting the reaction efficiency. Herein, the influence of furfural concentration on the electro-oxidation was investigated. As shown in Fig. 4, the furfural conversion gradually decreased with increasing furfural concentration. It is known that the amount of hydroxyl radicals formed was certain at a certain reaction time and applied potential, which means that the amount of furfural converted was certain, and thus the conversion decreased with increasing furfural concentration. In contrast, no obvious difference was found in the product (HFO and MA) selectivities and the FE. These results indicated that the furfural concentration affected its conversion rather than the product selectivities and the FE at a certain reaction time and applied potential.

### Reaction mechanism

**Scheme 2:**
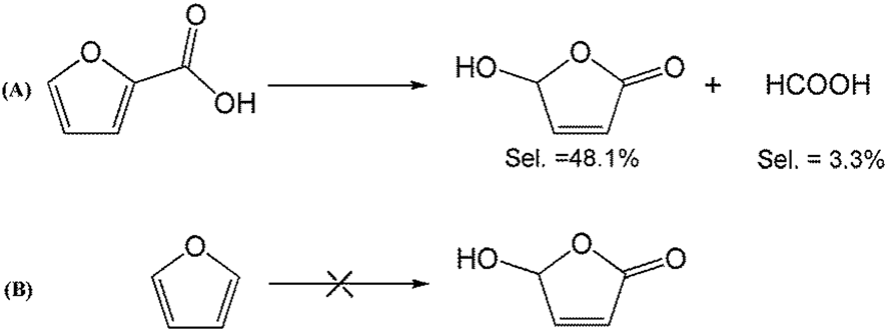
Control experiment using furoic acid and furan as the reactants.

**Scheme 3:**
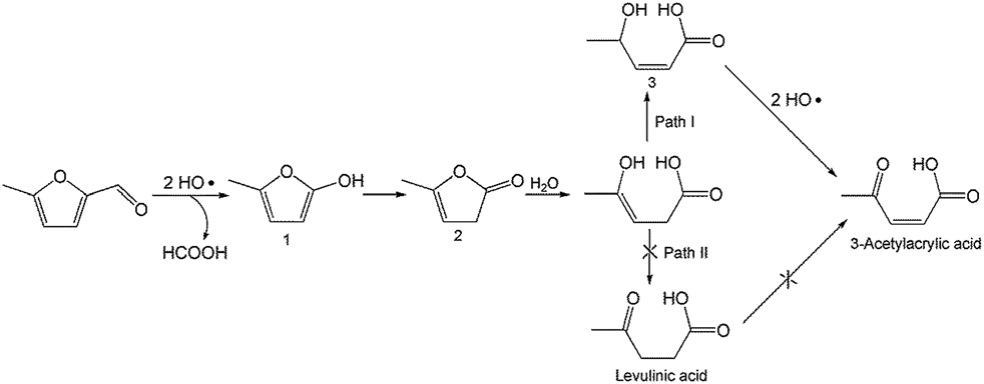
Control experiment using 5-methylfurfural as the reactant.

To better understand the reaction mechanism for furfural electro-oxidation to HFO, several control experiments were conducted under the above optimal reaction conditions. First, furoic acid was used as the reactant considering the fact that furfural could be easily oxidized to furoic acid. However, the HFO selectivity was only 48.1% from the electro-oxidation of furoic acid (Scheme 2A) along with a very low selectivity of HCOOH (3.3%) while the selectivity of HCOOH was above 95% using furfural as the reactant. These results indicated that furoic acid was not the main intermediate in the electrochemical oxidation of furfural to HFO. Second, no furan was generated in the process of furfural electro-oxidation, and the control experiment also showed that no HFO was formed when using furan as the reactant, suggesting that the formation of furan *via* the cleavage of the C–C bond (Scheme 2B) was not the reaction pathway. Third, in order to better obtain the reaction intermediates, 5-methylfurfural with a similar structure to furfural was used as the reactant (Scheme 3). Through ^1^H NMR and GC-MS analysis, HCOOH, 5-methyl-2(3*H*)-furanone (intermediate 2), intermediate 3, and 3-acetylacrylic acid were detected in the electrochemical oxidation of 5-methylfurfural (Fig. S22†). The above results indicated that 5-methyl-2(3*H*)-furanone (intermediate 2) was formed *via* the cleavage of the C–C bond to generate HCOOH and subsequent isomerization with the aid of the hydroxyl radicals generated by electrocatalysis. In one pathway (path I in Scheme 3), the generated 5-methyl-2(3*H*)-furanone could be converted to intermediate 3. Then, intermediate 3 could be further oxidized to 3-acetylacrylic acid. In another potential pathway (path II in Scheme 3), 5-methyl-2(3*H*)-furanone could be transformed into levulinic acid (not detected) *via* hydrolysis, and levulinic acid could not be oxidized to 3-acetylacrylic acid in a control experiment using levulinic acid as the reactant. The above results suggested that 5-methyl-2(3*H*)-furanone was transformed through the reaction pathway of path I (Scheme 3). Based on the above discussion, we could speculate that furfural could be converted into HFO by a similar route to path I.

**Scheme 4:**
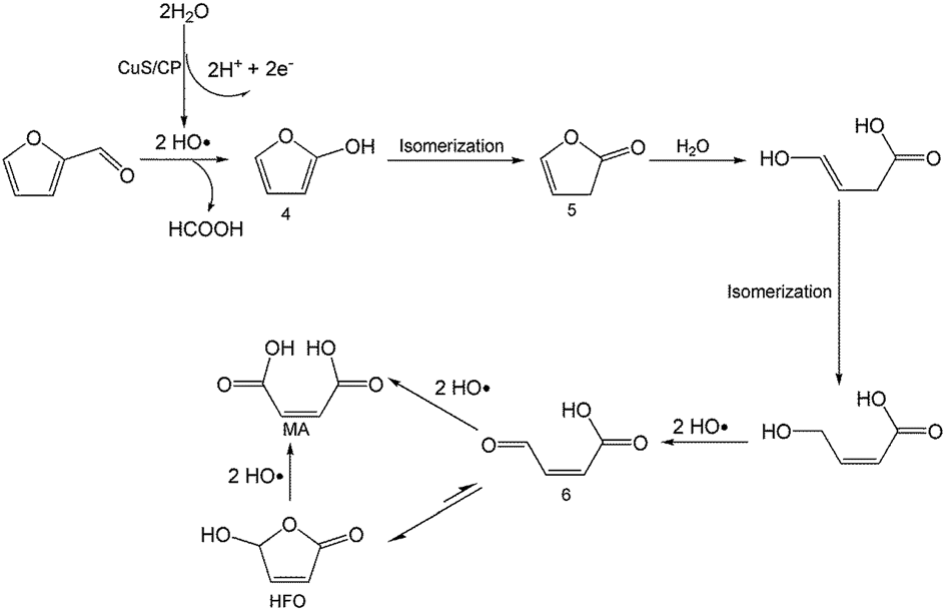
Possible reaction mechanism for electrochemical oxidation of furfural to HFO over the CuS/CP electrode in the [Et_3_NH]NO_3_–MeCN–H_2_O anolyte.

On the basis of the results obtained in this work and some reported studies,^11^,^22^–^27^ a reasonable mechanism was proposed for the electrochemical oxidation of furfural to HFO (Scheme 4). Initially, the hydroxyl radicals^22^–^27^ were generated from H_2_O by losing electrons on the surface of the CuS/CP electrode. Then, intermediate 4 and HCOOH were generated *via* the cleavage of C–C of furfural with the aid of the generated hydroxyl radicals, and the formed intermediate 4 was quickly converted into intermediate 5 *via* rearrangement. After the above steps, intermediate 6 (Fig. S23†) was formed through a pathway like path I (Scheme 3) using 5-methylfurfural as the reactant. Finally, HFO was generated through the isomerization of intermediate 6.^11^ In addition, HFO could be partially oxidized to MA by the formed hydroxyl radicals in the reaction process.

## Conclusions

In conclusion, electrochemical oxidation of furfural to HFO was achieved for the first time by employing metal chalcogenides (*i.e.*, CuS, ZnS, PbS, CdS, WS_2_, and MoS_2_) as electrocatalysts and H_2_O as the oxygen source under ambient conditions. Among the used electrodes, the CuS/CP electrode prepared in this work showed the best performance, and high HFO selectivity (83.6%) could be obtained with a furfural conversion of 70.2% in a ternary anolyte consisting of [Et_3_NH]NO_3_ (1.8 wt%), MeCN, and H_2_O (12.5 wt%). Moreover, the prepared CuS/CP electrode showed excellent long-term stability for the electrochemical oxidation of furfural to HFO. Mechanism investigation indicated that HFO was generated through multistep reactions, including the cleavage of C–C bonds, subsequent ring opening and oxidation, and intramolecular isomerization. This work opens an avenue to produce high value-added HFO *via* electrochemical oxidation of renewable furfural using H_2_O as the oxygen source. We believe that the electrocatalytic strategy can also be used to explore routes for efficient transformation of other furan derivatives into valuable chemicals.

## Experimental

### Materials

Triethylammonium nitrate ([Et_3_NH]NO_3_, 98%) was purchased from the Centre for Green Chemistry and Catalysis, Lanzhou Institute of Chemical Physics, Chinese Academy of Sciences. Polyethylene glycol (PEG-200, 99%), thiourea (99%), molybdenum disulfide (99%), and copper oxide (CuO, 97%) were supplied by J&K Scientific Co., Ltd. Tungsten sulphide (99%) was purchased from Adamas Reagent Co., Ltd. Copper nitrate trihydrate (99%), lead acetate trihydrate (99%) and zinc nitrate hexahydrate (99%) were obtained from Sinopharm Chemical Reagent Co., Ltd. Nafion D-521 dispersion (5% w/w in water and 1-propanol, ≥0.92 meg per g exchange capacity), Nafion N-117 membranes (0.180 mm thick, ≥0.90 meg per g exchange capacity), copper sulfide (99.8%), cadmium sulphide (98%) and Toray carbon paper (CP, TGP-H-60, 19 × 19 cm) were purchased from Alfa Aesar China Co., Ltd.

### Preparation of metal chalcogenides (MC)

The procedures are discussed taking the preparation of CuS as an example. First, a deep eutectic solvent consisting of thiourea and PEG-200 with a molar ratio of 1 : 2 was prepared on the basis of the reported method.^37^ Then, Cu(NO_3_)_2_·3H_2_O (1.0 g) was dissolved into the prepared deep eutectic solvent (40.0 g) under stirring. After that, the mixture was transferred into a stainless steel reactor with a Teflon coating, and the reactor was maintained at 180 °C for 24 h. Finally, the prepared CuS was washed with ethanol and water three times, and was dried under vacuum at 40 °C for 24 h. The preparation process of PbS and ZnS was similar to that for preparing CuS. The preparation conditions for the synthesis of PbS: Pb(CH_3_COO)_2_·3H_2_O (3.0 g), solvent (70.0 g), and reaction temperature (180 °C) for 24 h. The preparation conditions for the synthesis of ZnS: Zn(NO_3_)_2_·6H_2_O (1.0 g), solvent (40.0 g), and reaction temperature (180 °C) for 12 h.

### Preparation of electrodes

The prepared metal chalcogenides (MC, 12.0 mg) were suspended in 2 mL ethanol with 40 μL Nafion D-521 dispersion (5 wt%) to form a homogeneous ink with the assistance of ultrasound. After that, 1 mL of the ink was spread onto the carbon paper (CP) surface (1 cm × 1 cm), and then dried at room temperature. Finally, the electrodes were obtained and are denoted as MC/CP.

### Characterization

Transmission electron microscopy (TEM) JEOL-1011 with an accelerating voltage of 120 kV was used for TEM characterization. The scanning electron microscopy (SEM) experiment was conducted on a Hitachi S-4800 scanning electron microscope operated at 15 kV. Powder X-ray diffraction (XRD) patterns were collected on a Rigaku D/max-2500 X-ray diffractometer using Cu Kα radiation (*λ* = 0.154 nm). X-ray photoelectron spectroscopy (XPS) measurements were carried out on an ESCALAB 220i-XL spectrometer. ^1^H NMR spectra were recorded on a Bruker Avance III HD 400 MHz NMR spectrometer.

### Linear sweep voltammetry (LSV) measurements

All the electrochemical experiments in this work were conducted using an electrochemical workstation (CHI 660E, Shanghai CH Instruments Co., China). An H-type cell separated by a Nafion 117 membrane was used for the linear sweep voltammetry (LSV) measurements. There were three electrodes in the system including a working electrode (MC/CP), a platinum gauze auxiliary electrode, and an Ag/Ag^+^ (0.01 M AgNO_3_ in 0.1 M TBAP-MeCN) reference electrode. Prior to the experiment, the air in the electrolyte was removed by bubbling it with N_2_ for 30 minutes. The LSV measurement was performed in the potential range of 0.3 to 1.9 V *vs.* Ag/Ag^+^ at a sweep rate of 20 mV s^–1^. The process was carried out under slight magnetic stirring.

### Electrochemical impedance spectroscopy (EIS)

The EIS experiment was conducted using a single compartment cell with three electrodes, namely, a working electrode, a platinum gauze auxiliary electrode, and an Ag/Ag^+^ (0.01 M AgNO_3_ in 0.1 M TBAP-MeCN) reference electrode. EIS spectra were collected in potentiostatic mode at an open circuit potential of 100 kHz to 100 mHz with an amplitude of 5 mV. The EIS data were fitted using ZSimpwin software.

### Electrocatalytic oxidation of furfural and product analysis

Electrochemical oxidation of furfural was performed in a typical H-type cell at room temperature, which was similar to that used for electrochemical reduction of CO_2_.^32^ The anodic and cathodic electrolytes were [Et_3_NH]NO_3_–MeCN–H_2_O and aqueous H_2_SO_4_ solution (0.2 M), respectively, and the amount of electrolyte in each chamber was 5.6 g in all experiments. Prior to electrolysis, N_2_ was bubbled through the anolyte for 30 min under stirring. Then, furfural (1 mmol) was added to the anolyte, and the electrochemical reaction was started at a desired applied potential. After the reaction was conducted for a suitable reaction time, the liquid product was analyzed by ^1^H NMR (Bruker Avance III 400 HD spectrometer) in D_2_O. The gaseous product was analyzed with a gas chromatograph (GC, HP 4890D) equipped with a TCD detector using helium as the internal standard. The conversion and selectivity of the reaction were calculated using NMR and GC analysis.

## Conflicts of interest

There are no conflicts to declare.

## Supplementary Material

Supplementary information
