# Supplementary material for: An electrocatalytic route for transformation of biomass-derived furfural into 5-hydroxy-2(5H)-furanone
Source: Chem Sci. 2019 Mar 25;10(17):4692–8. doi: 10.1039/c9sc00322c (PMC6495687; doi:10.1039/c9sc00322c)
Supplement: Supplementary file 1 [file SC-010-C9SC00322C-s001.pdf]

Electronic Supplementary Material (ESI) for Chemical Science.
This journal is © The Royal Society of Chemistry 2019

Electrocatalytic route for transformation of biomass-derived furfural to 5-hydroxy-2(5H)-furanone

Haoran Wu,[a,b] Jinliang Song,*[a] Huizhen Liu,[a,b] Zhenbing Xie,[a,b] Chao Xie,[a,b] Yue Hu,[a,b] Xin Huang,[a,b] Manli Hua,[a,b] and Buxing Han*[a,b]

[a]Beijing National Laboratory for Molecular Science, CAS Key Laboratory of Colloid and Interface and Thermodynamics, CAS Research/Education Center for Excellence in Molecular Sciences, Institute of Chemistry, Chinese Academy of Sciences, Beijing 100190, China.

[b]School of Chemistry and Chemical Engineering, University of Chinese Academy of Sciences, Beijing 100049, China.

E-mails: songjl@iccas.ac.cn; hanbx@iccas.ac.cn.

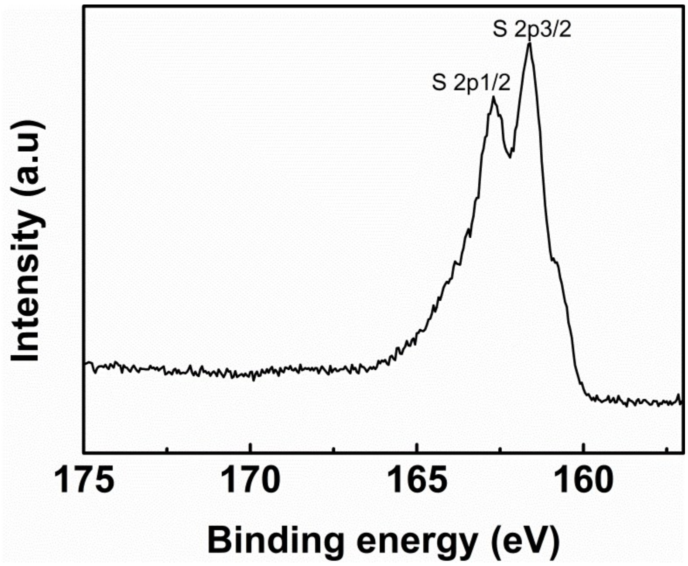

Fig. S1. XPS of S 2p in the prepared CuS.

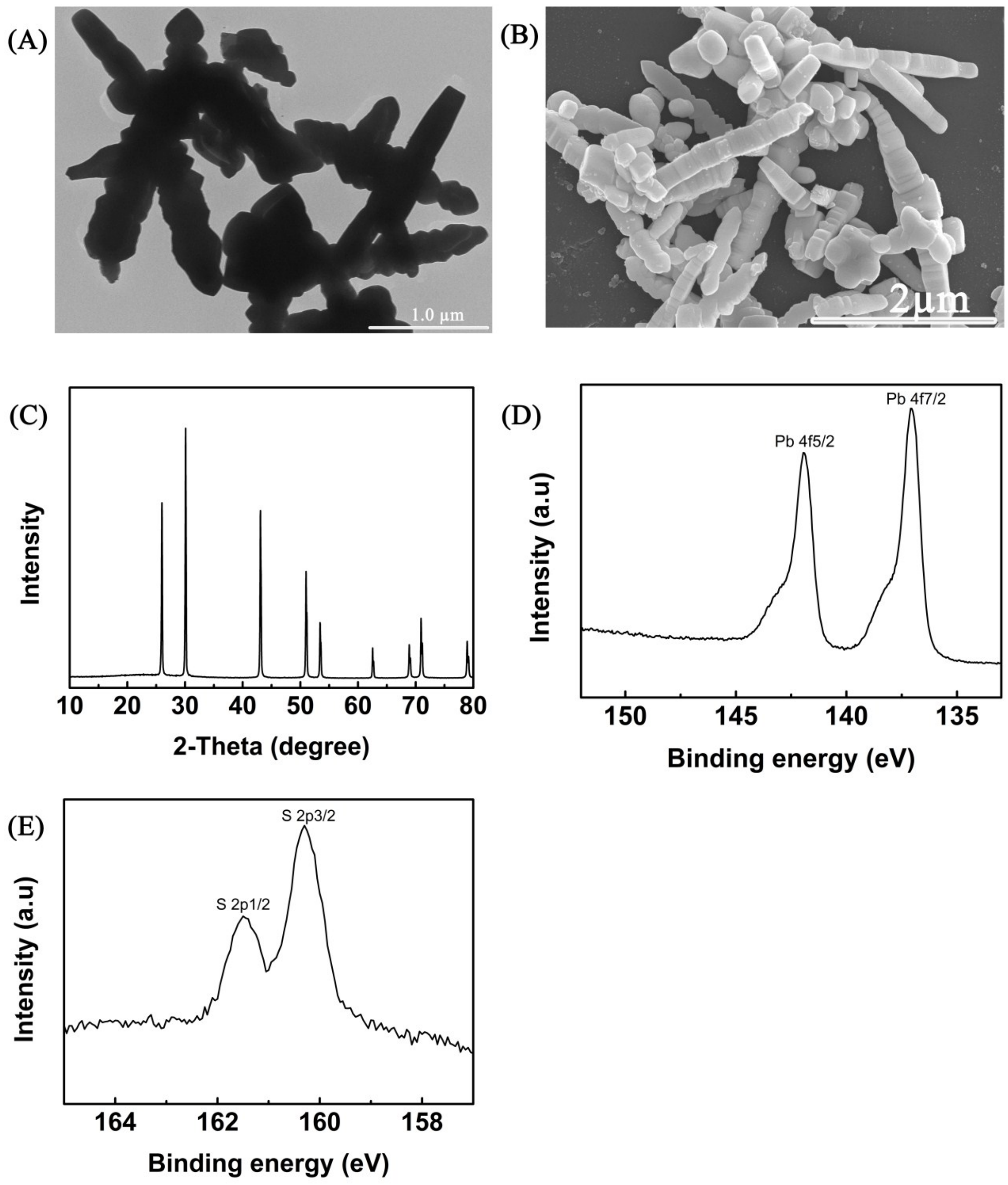

Fig. S2. Characterization of the prepared PbS. TEM image (A), SEM image (B), XRD (C), XPS of Pb 4f (D), and S 2p (E).

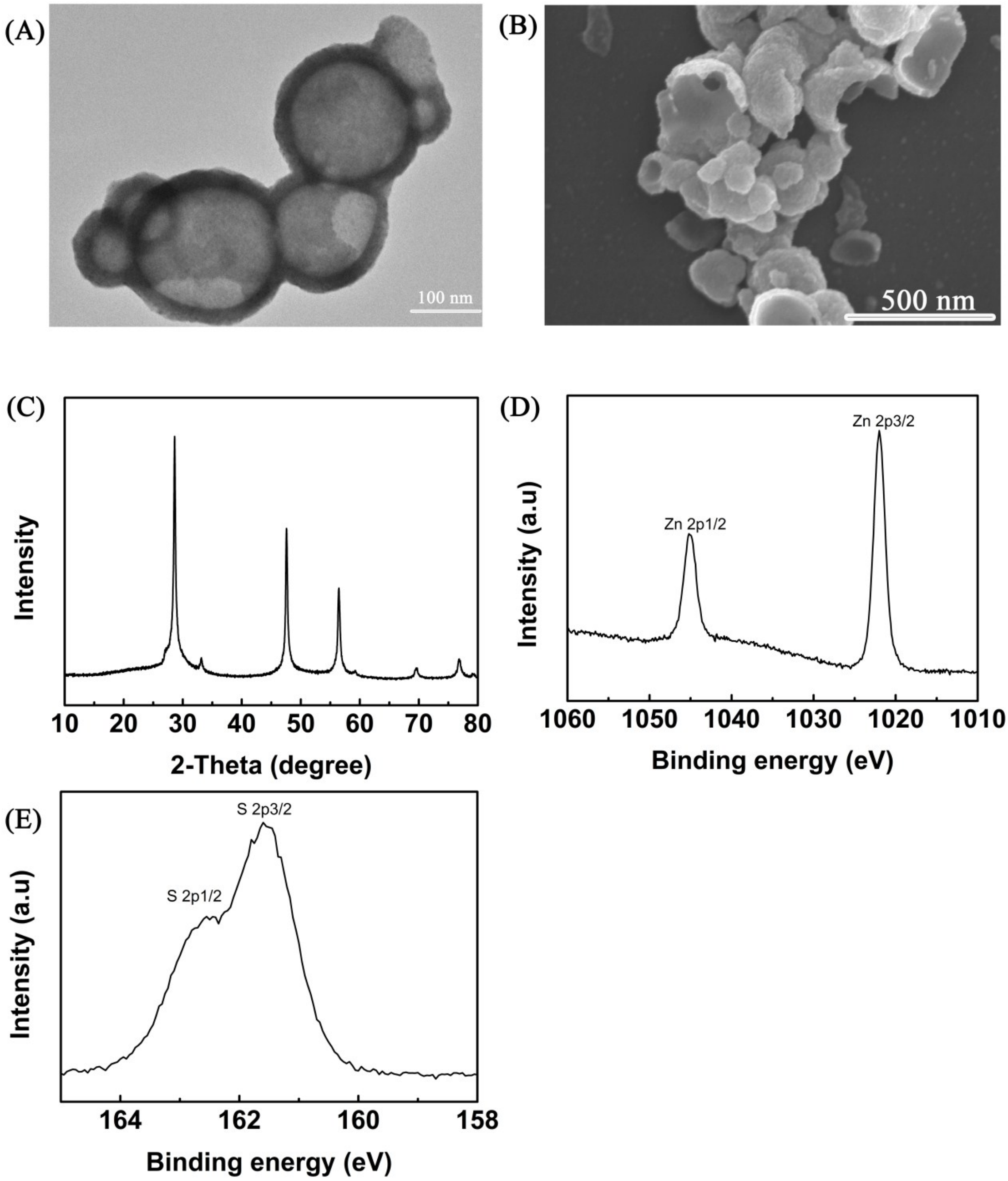

Fig. S3. Characterization of the prepared ZnS. TEM image (A), SEM image (B), XRD (C), XPS of Zn 2p (D), and S 2p (E).

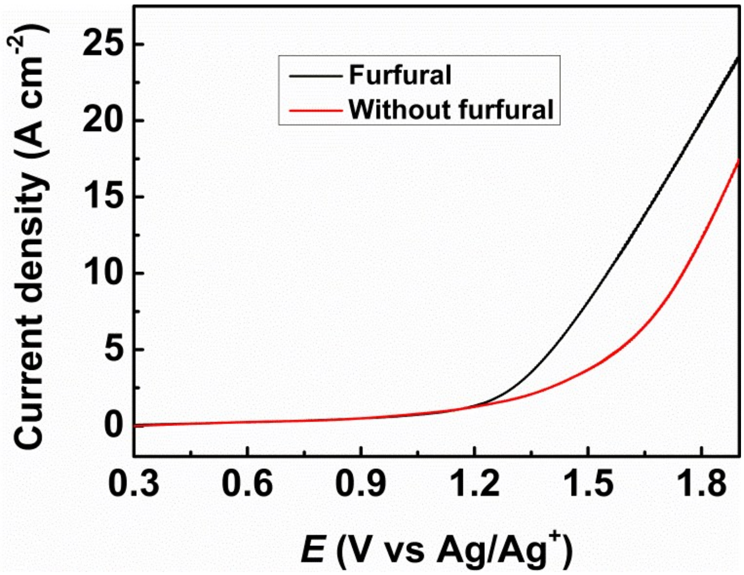

Fig. S4. LSV measurements using the prepared PbS/CP electrode for the electrochemical oxidation of furfural in the electrolyte of $[Et_3NH]NO_3$ (1.8 wt%)-MeCN-H_2O (12.5 wt%).

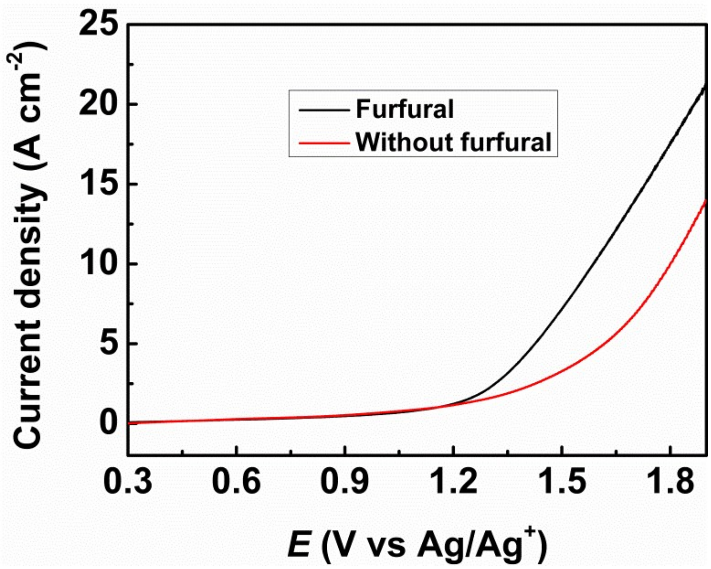

Fig. S5. LSV measurements using the prepared ZnS/CP electrode for the electrochemical oxidation of furfural in the electrolyte of $[Et_3NH]NO_3$ (1.8 wt%)-MeCN-H_2O (12.5 wt%).

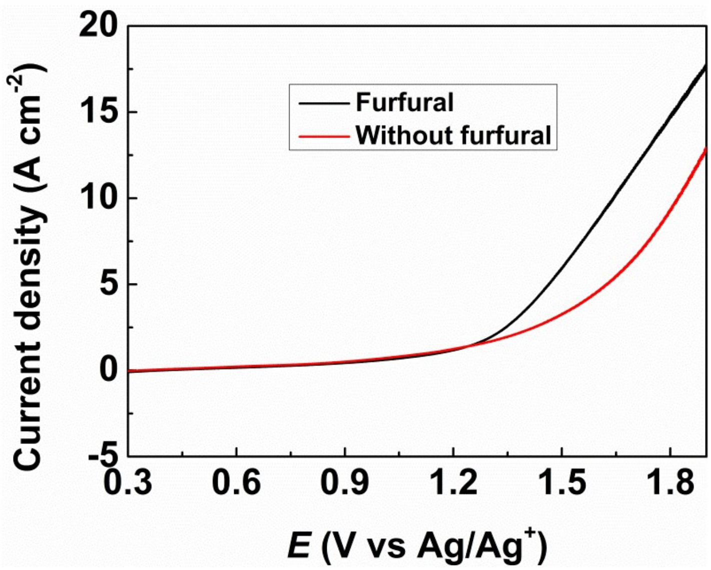

Fig. S6. LSV measurements using the prepared WS_2/CP electrode for the electrochemical oxidation of furfural in the electrolyte of $[Et_3NH]NO_3$ (1.8 wt%)-MeCN-H_2O (12.5 wt%).

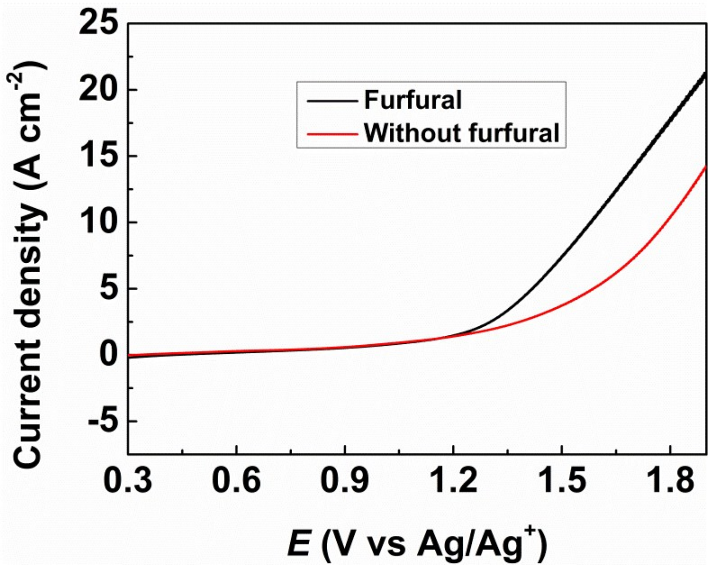

Fig. S7. LSV measurements using the prepared MoS_2/CP electrode for the electrochemical oxidation of furfural in the electrolyte of $[Et_3NH]NO_3$ (1.8 wt%)-MeCN-H_2O (12.5 wt%).

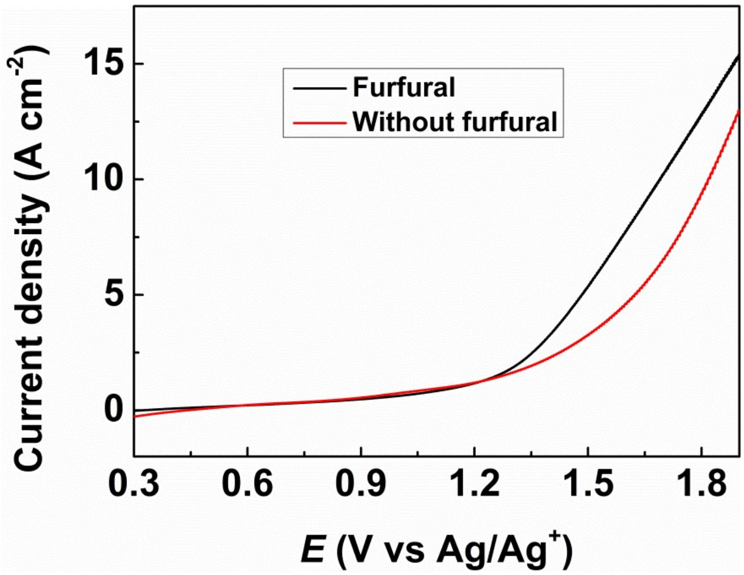

Fig. S8. LSV measurements using the prepared CdS/CP electrode for the electrochemical oxidation of furfural in the electrolyte of $[Et_3NH]NO_3$ (1.8 wt%)-MeCN-H_2O (12.5 wt%).

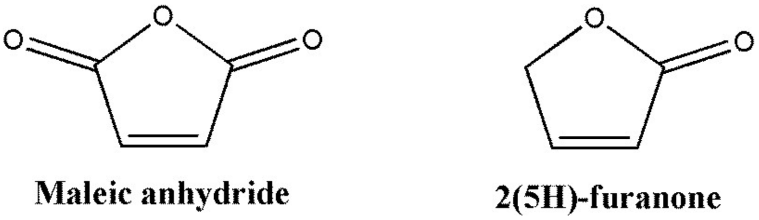

Scheme S1. The structures of maleic anhydride and 2(5H)-furanone.

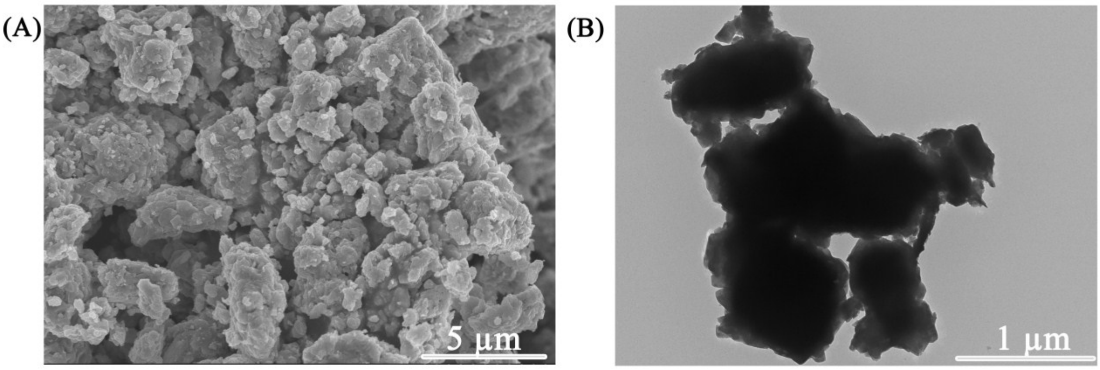

Fig. S9. Characterization of commercial CuS. SEM (A) and TEM (B) images.

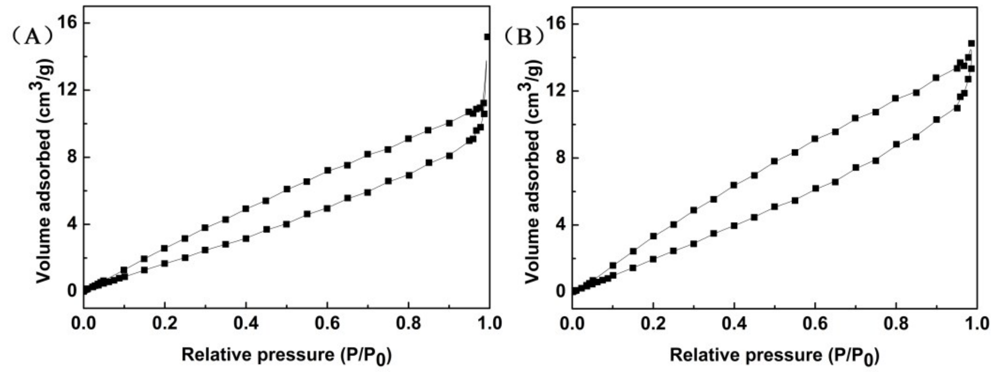

Fig. S10. N_2 adsorption-desorption isotherm of the commercial CuS (A) and prepared CuS (B)

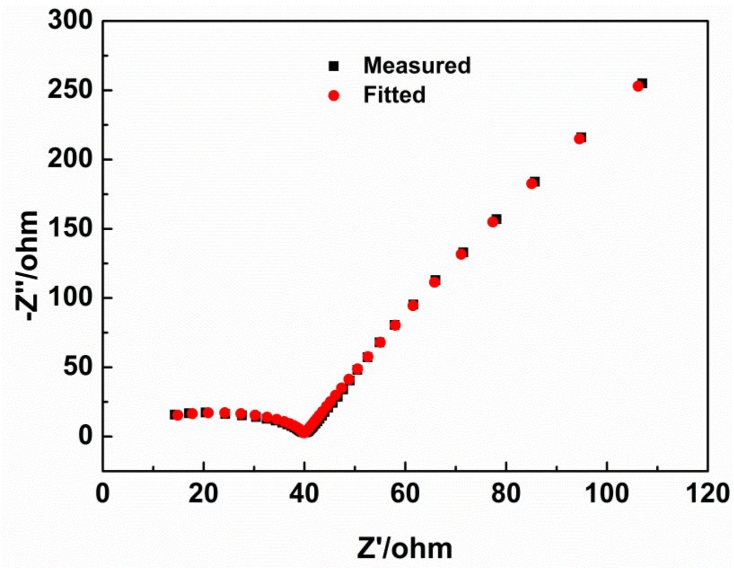

Fig. S11. The measured and fitted EIS spectra of CuS/CP in $[Et_3NH]NO_3$ (1.8 wt%)-MeCN-H_2O (12.5 wt%) electrolyte in the presence of furfural.

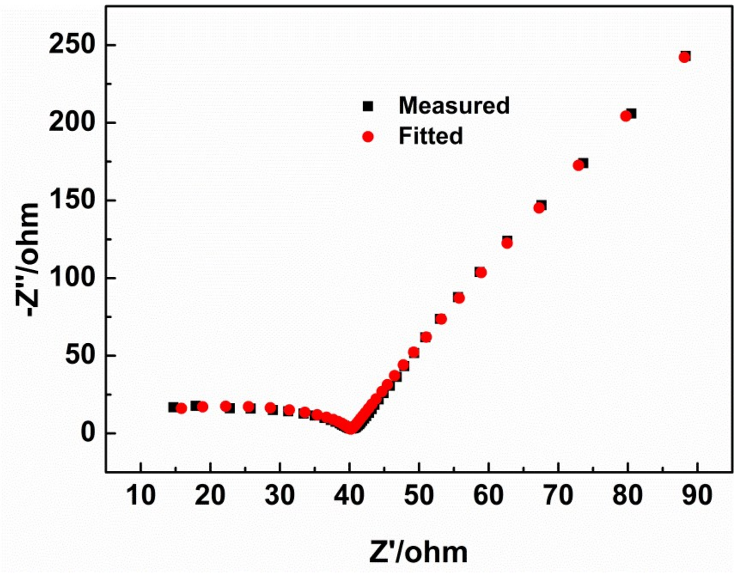

Fig. S12. The measured and fitted EIS spectra of PbS/CP in $[Et_3NH]NO_3$ (1.8 wt%)-MeCN-H_2O (12.5 wt%) electrolyte in the presence of furfural.

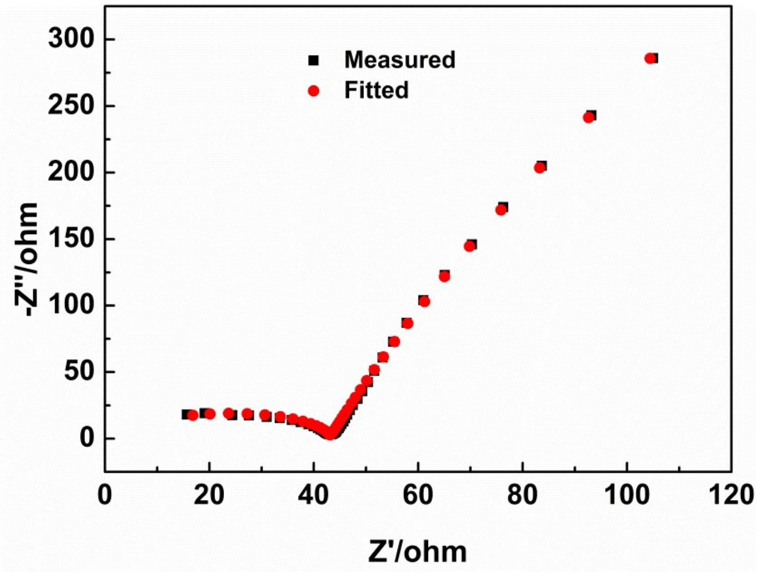

Fig. S13. The measured and fitted EIS spectra of ZnS/CP in $[Et_3NH]NO_3$ (1.8 wt%)-MeCN-H_2O (12.5 wt%) electrolyte in the presence of furfural.

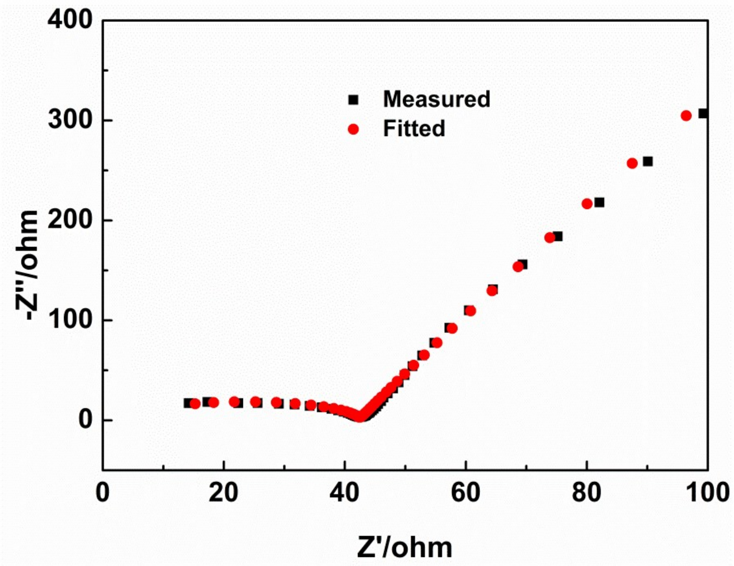

Fig. S14. The measured and fitted EIS spectra of CuO/CP in $[Et_3NH]NO_3$ (1.8 wt%)-MeCN-H_2O (12.5 wt%) electrolyte in the presence of furfural.

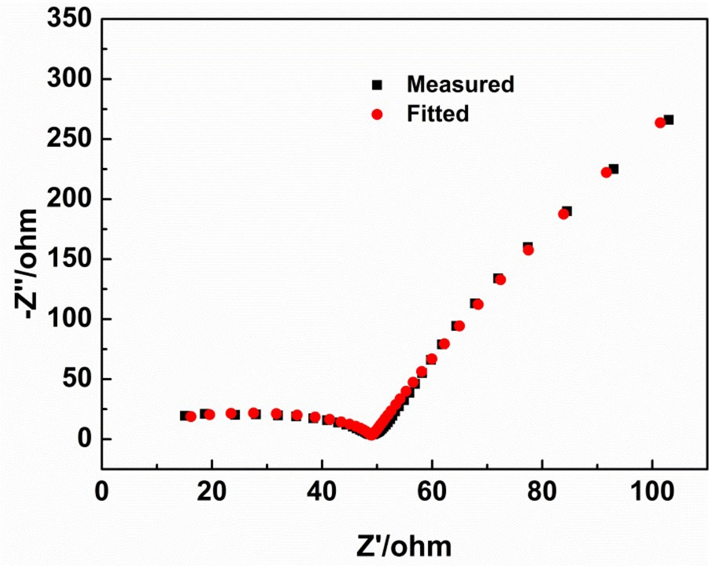

Fig. S15. The measured and fitted EIS spectra of CdS/CP in $[Et_3NH]NO_3$ (1.8 wt%)-MeCN-H_2O (12.5 wt%) electrolyte in the presence of furfural.

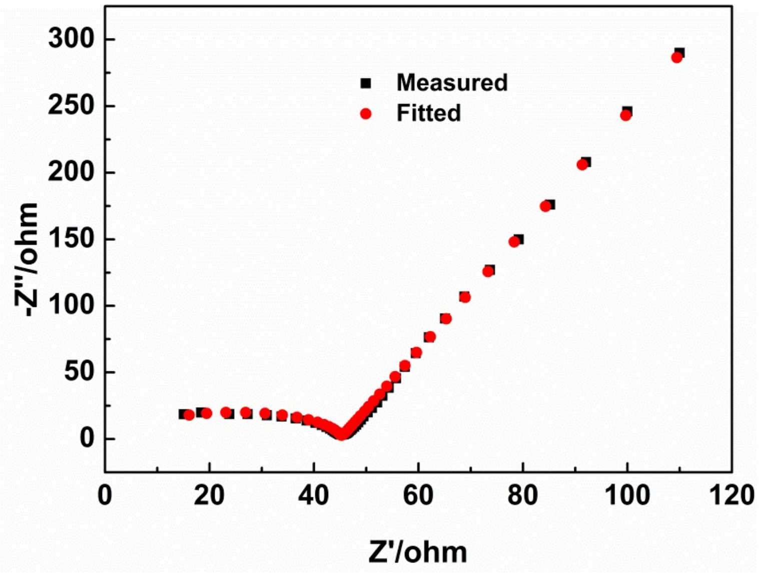

Fig. S16. The measured and fitted EIS spectra of MoS_2/CP in [Et_3NH]NO_3 (1.8 wt%)-MeCN-H_2O (12.5 wt%) electrolyte in the presence of furfural.

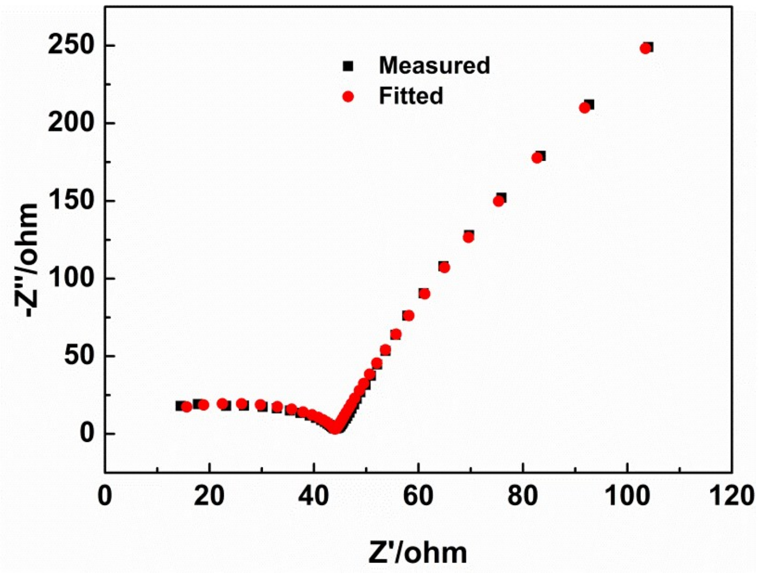

Fig. S17. The measured and fitted EIS spectra of WS_2/CP in [Et_3NH]NO_3 (1.8 wt%)-MeCN-H_2O (12.5 wt%) electrolyte in the presence of furfural.

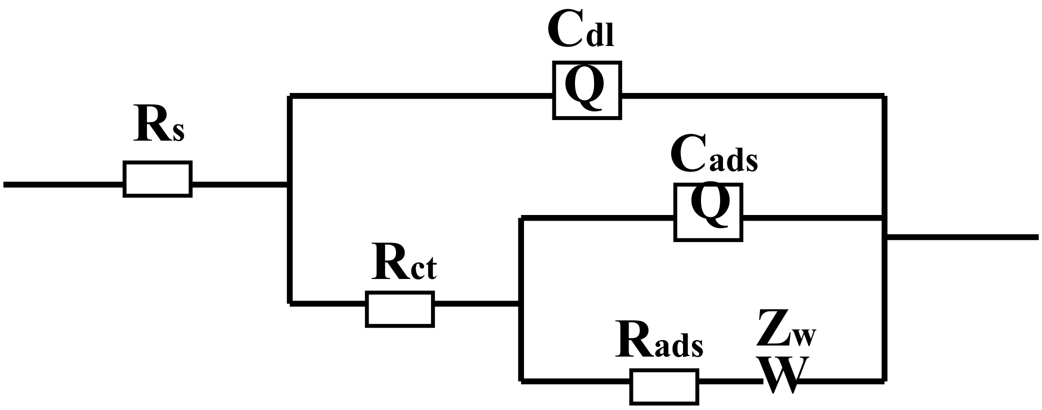

Fig. S18. Electrical equivalent circuit for fitting the measured impedance data. R_s, R_{ct}, C_{dl}, C_{ads}, Z_w, and R_{ads} represent solution resistance, electron transfer resistance, double layer capacitance, surface adsorption capacitance, Warburg impedance, and surface adsorption resistance, respectively.

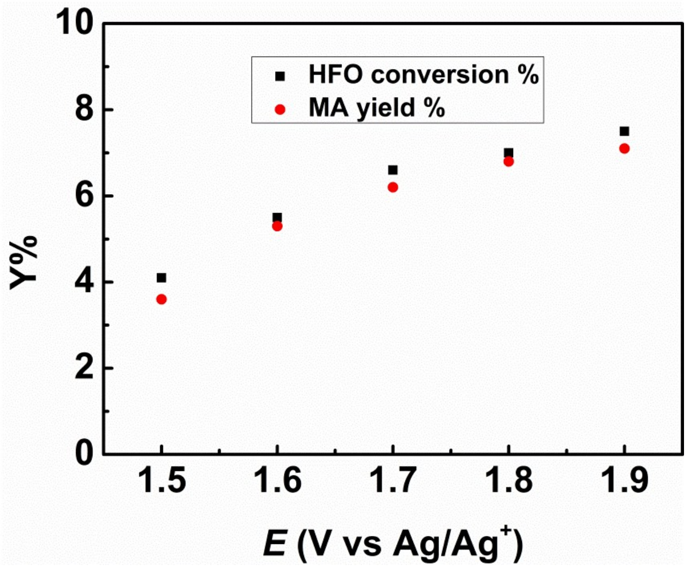

Fig. S19. Effect of applied potential on the electrochemical oxidation of HFO over CuS/CP electrode in the electrolyte (5.6 g) of $[Et_3NH]NO_3$ (1.8 wt%)-MeCN-H_2O (12.5 wt%) with 1 mmol of HFO for 7 h.

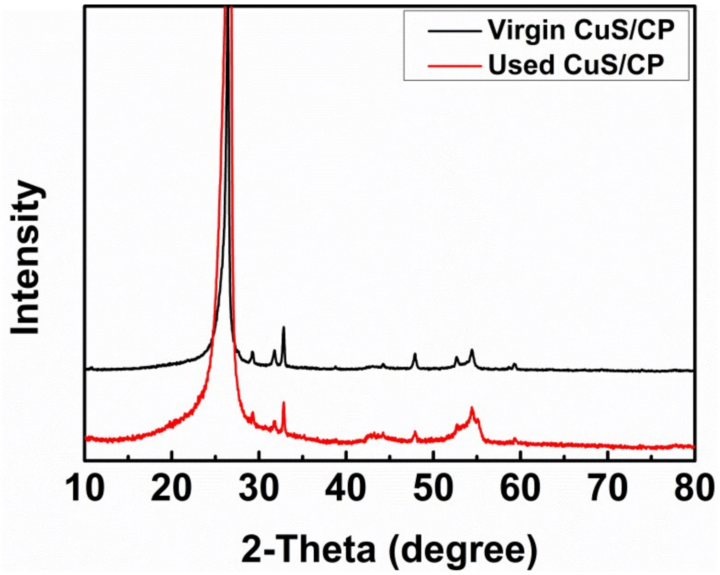

Fig. S20. XRD patterns of the virgin and used CuS/CP (24 h) electrode.

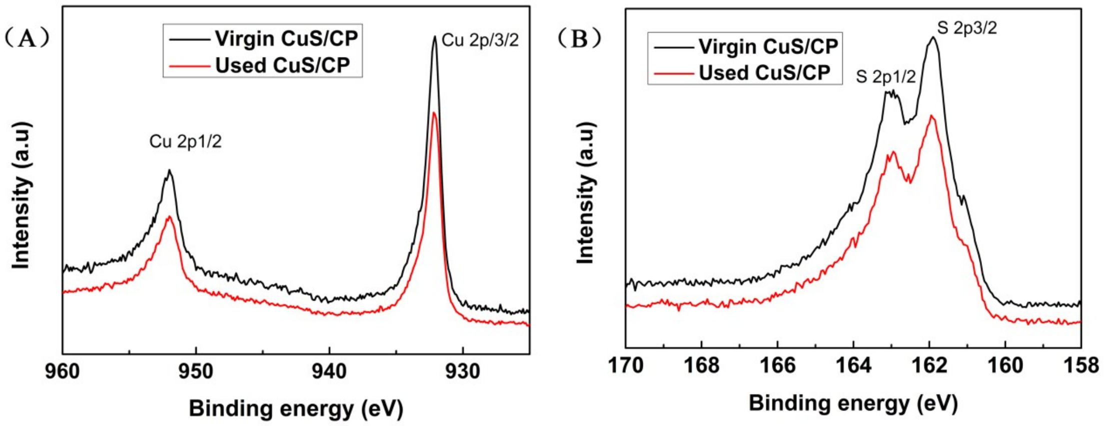

Fig. S21. XPS of the virgin and used CuS/CP (24 h) electrode.

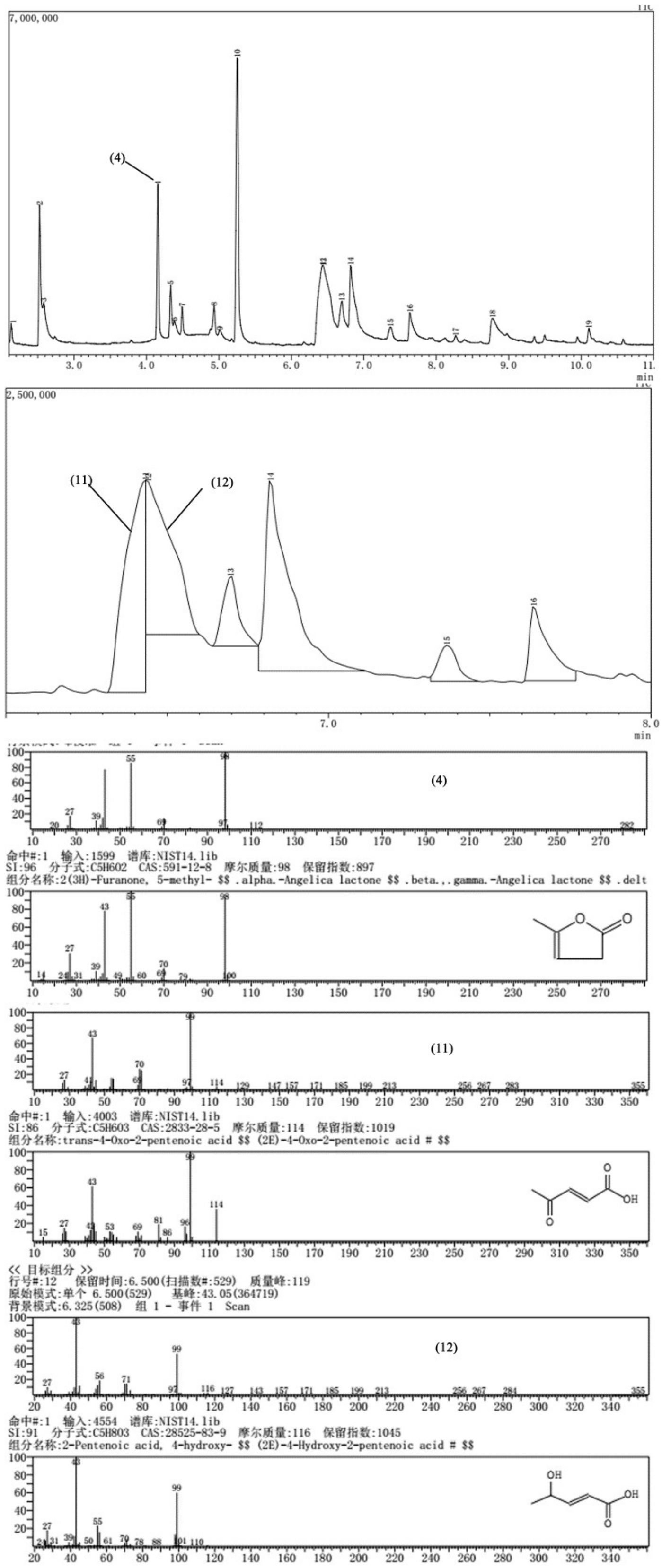

Fig. S22. GC-MS spectra of electrolyte after electrolysis for the electrochemical oxidation of 5-methylfurfural using CuS/CP in $[Et_3NH]NO_3$ (1.8 wt%)-MeCN-H_2O (12.5 wt%) at 1.6 V *vs.* Ag/Ag^+.

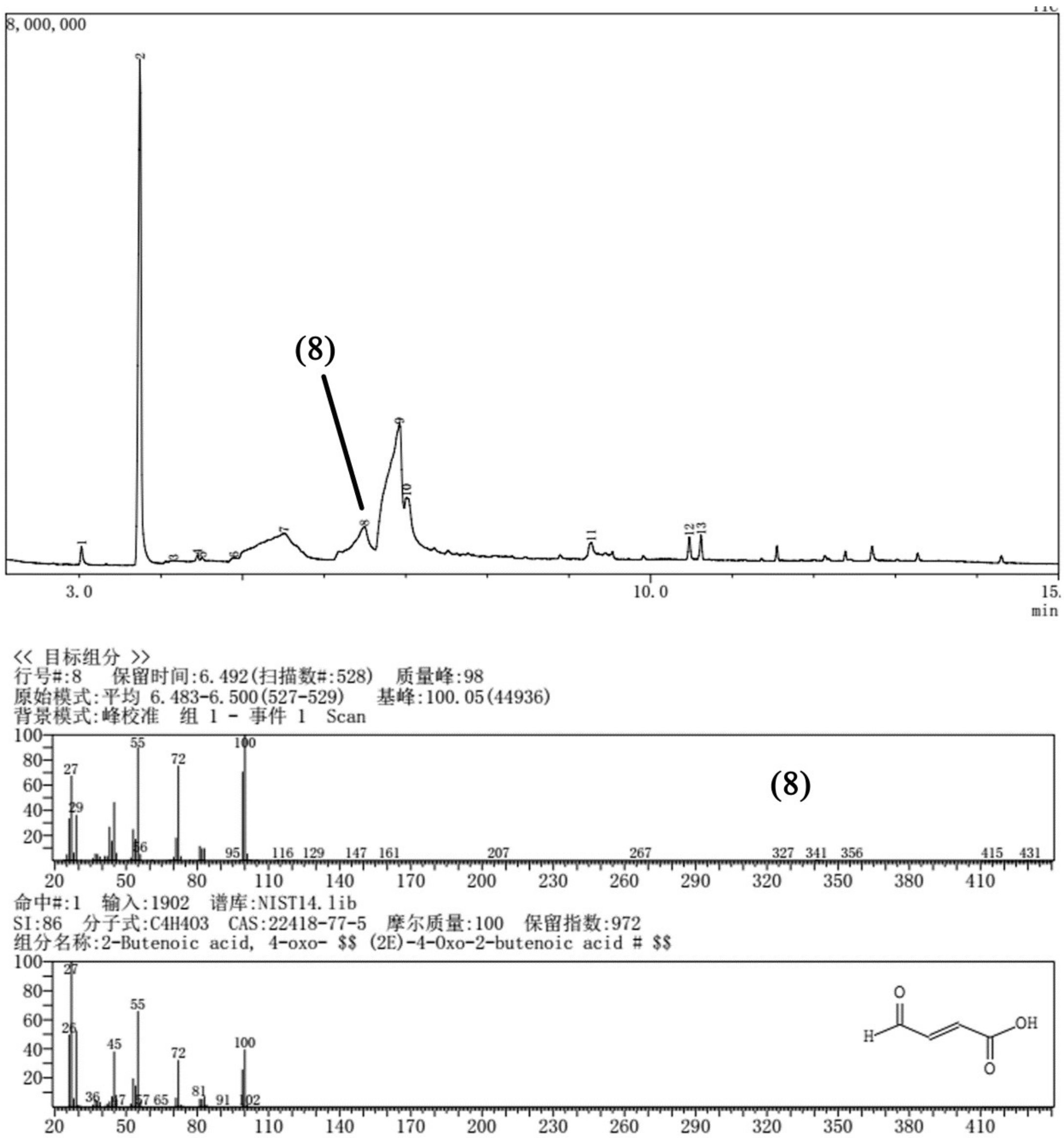

Fig. S23. GC-MS spectra of electrolyte after electrolysis for the electrochemical oxidation of furfural using CuS/CP in $[Et_3NH]NO_3$ (1.8 wt%)-MeCN-H_2O (12.5 wt%) at 1.6 V *vs.* Ag/Ag^+.

Table S1 Parameter values of the equivalent circuit model.

Entry	Electrode	R_s (Ω cm^{-2})	R_c (Ω cm^{-2})	Cdl (×10^{-2} μF cm^{-2})
1	PbS/CP	5.045	35.02	6.796
2	ZnS/CP	5.174	37.86	6.294
3	CdS/CP	5.344	43.54	6.346
4	MoS_2/CP	4.993	40.80	6.396
5	WS_2/CP	4.938	39.01	6.624
6	CuS/CP	5.240	34.51	7.405
7	CuO/CP	5.215	37.17	7.022

Table S2. The conductivity of $[Et_3NH]NO_3$-CH_3CN- H_2O (12.5 wt%) electrolyte.

Entry	$[Et_3NH]NO_3$ (wt%)	Conductivity (ms cm^{-1})
1	0.9	4.28
2	1.8	6.81
3	3.6	10.84
4	5.4	14.69
5	7.1	18.13
6	8.9	20.50
